# Traumatic Cerebral Myiasis in the Elderly: The First Reported Case From India

**DOI:** 10.7759/cureus.48484

**Published:** 2023-11-08

**Authors:** Sankalp Yadav, Gautam Rawal, Madhan Jeyaraman

**Affiliations:** 1 Medicine, Shri Madan Lal Khurana Chest Clinic, Moti Nagar, New Delhi, IND; 2 Respiratory Medical Critical Care, Max Super Speciality Hospital, Saket, New Delhi, IND; 3 Orthopaedics, ACS Medical College and Hospital, Dr. MGR Educational and Research Institute, Chennai, IND

**Keywords:** traumatic cerebral myiasis, myiasis, larvae, cerebral infestation, brain

## Abstract

A medical condition known as myiasis, or maggot infestation, occurs when fly larvae eat living tissue and continue to grow inside of it. Tropical and subtropical areas are where it is most commonly seen. Human myiasis is more prevalent among individuals who have close contact with domestic animals and those living in unhygienic conditions. Traumatic cerebral myiasis is a rare clinical condition in humans with only two such cases documented in the literature. It is brought on by a parasite infestation, also referred to as a maggot infestation of dipteran larvae, on an untended open wound due to trauma. In this report, we present an exceedingly rare case of cerebral myiasis in a 54-year-old Indian male, the first report from India, occurring as a result of trauma. Wound debridement with maggots removal and antibiotic administration was done. However, the patient was lost to follow-up.

## Introduction

According to Zumpt, myiasis is the term used to describe the infestation of live humans and vertebrate animals by larvae of Diptera species [1]. For a certain amount of time, these larvae devour the host's tissues, body fluids, or consumed food [2]. Although it is not a common clinical illness, human myiasis is more frequently seen in tropical and subtropical areas [3]. Myiasis was initially described in writing in 1840 by Hope [1,3]. These larvae have the capability to infect the skin, necrotic tissues, and natural cavities within living individuals [3].

Cerebral myiasis, a condition in which fly larvae infest the brain, is exceptionally uncommon, and its occurrence involving a substantial portion of brain tissue can lead to severe symptoms [4]. In order to shed light on this infrequently reported ailment, we report a singular instance of cerebral myiasis in a 54-year-old male from New Delhi, India.

## Case presentation

A 54-year-old homeless Indian male was found on the roadside and was brought to the emergency department with a large wound on the scalp and a maggot infestation. On detailed history, he was destitute with a history of falls resulting in an open wound for two months.

The patient's vital signs were within the normal range, but he had a fever (100.6°F). A complete blood cell count showed a leukocyte count of 14.9 K/μL, with 92% neutrophils. The rest of his laboratory work was insignificant.

**Figure 1:**
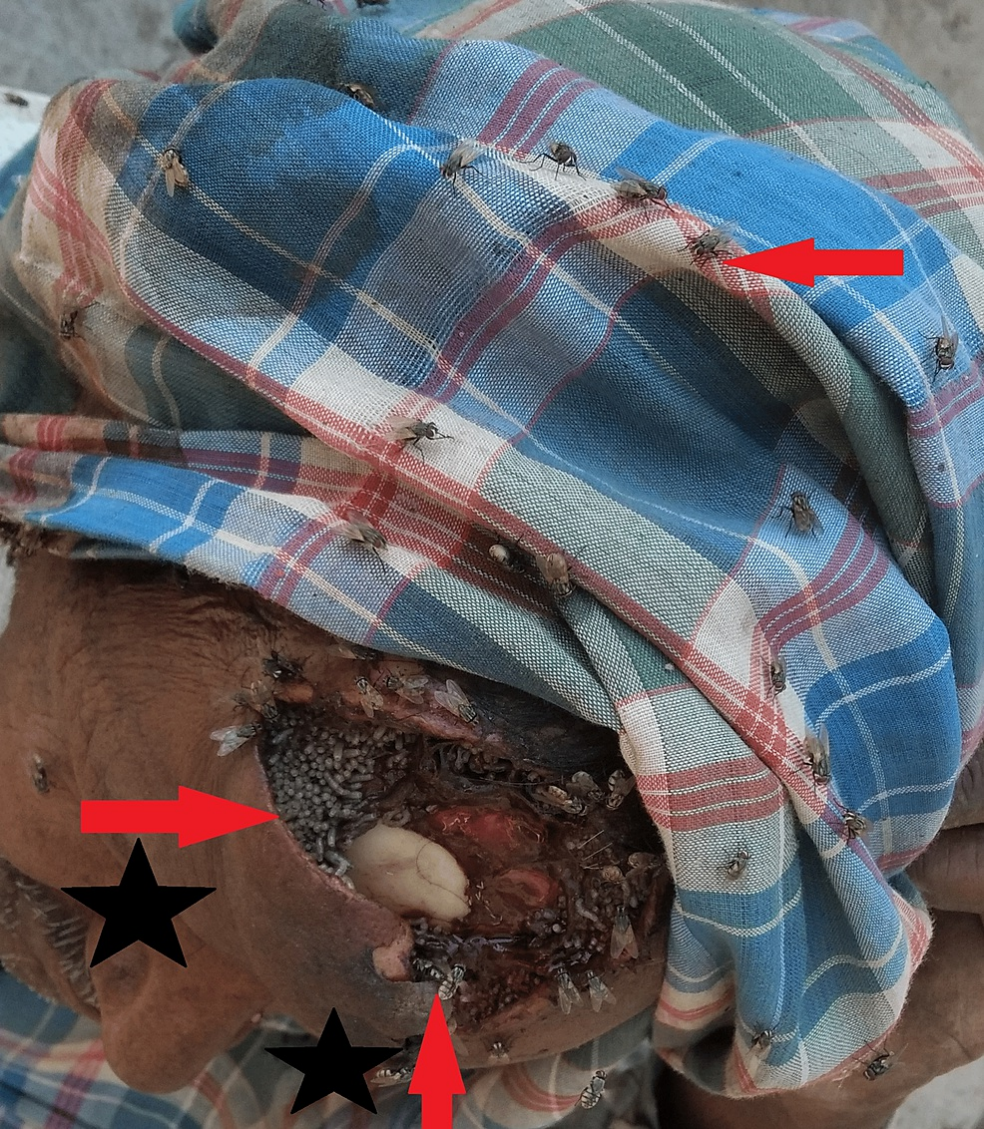
Gross image showing the wound, maggots, and the flies

During the physical examination, a large frontal scalp and cranial wound, measuring 23 cm × 19 cm, was observed. The skin surrounding the erosion exhibited hypertrophy, redness, and an exposed dura mater. At the center of the defect, a cluster of nearly 100 maggots and flies were seen, and there was no visible cerebrospinal fluid leakage (Figure 1).

A mental health evaluation was unremarkable for any cognitive impairment. For cleaning and debridement of the wound, the patient was taken to the operating room. Approximately 120 maggots were carefully removed using forceps, turpentine oil, and suction.

The tissues and visible bone were meticulously cleaned and debrided. Following the procedure, a two-week course of a broad-spectrum antibiotic, ampicillin and sulbactam, was administered. Daily aseptic dressings were advised to facilitate secondary wound healing. No bacterial growth was detected from the tissue culture samples collected during the intraoperative procedure. Samples of the maggots were sent to the zoology department for examination. Subsequently, the maggots were identified as *Musca domestica nebulo*, commonly known as the housefly. The patient was advised to return for a follow-up visit, but he failed to do so.

## Discussion

Traumatic cerebral myiasis is an extremely uncommon condition, with only two documented cases globally. The majority of these cases have been identified following surgical procedures. In India, myiasis is frequently attributed to the common housefly, *Musca domestica nebulo*, and is particularly prevalent during the summer and rainy seasons, often in areas with subpar hygiene and sanitation [5]. A fly's life cycle starts with the egg state and continues through the larval, pupal, and adult stages, which are all eventually exhibited [3,5].

Fly larvae that infest open wounds owing to either obligatory or facultative parasitic conditions may trigger wound myiasis [3]. Housefly (*Musca domestica*) larvae are usually found in decomposing waste and manure and primarily feed on decaying matter. Most species within the Sarcophagidae family that lead to myiasis lay their eggs or larvae in specific predisposing locations on the host, such as areas with wounds, necrosis, or bacterial contamination [1].

Several factors predispose individuals to cutaneous myiasis, including advanced age, unfavorable social conditions, inadequate personal hygiene, poor general health, immunocompromised status, psychiatric illnesses/mental retardation, immobility, diabetes mellitus, alcoholism, vascular occlusive disease, ulcerated lesions, bacterial wound infections, infected dermatitis, travel to regions with endemic myiasis, and contact with livestock [5]. Our case also presented with wound myiasis and exhibited characteristics such as lower socioeconomic status, suboptimal personal hygiene, and contact with livestock. Moreover, there was a lack of awareness as well as education, which were the major contributing factors.

Based on where it occurs, myiasis is divided into two categories: main and secondary. While fly eggs are deposited into an already-existing skin ulcer in the secondary form of myiasis, in the primary form, larvae penetrate the skin via a wound that has been punctured. Secondary myiasis has been linked to various benign or malignant traumatic or dermatological conditions, primarily stemming from insufficient wound management [6].

As demonstrated in our illustration, the frontal lobe appears to be the preferred location for the infestation. The frontal lobe is statistically most probable to be affected in any general process because it is the largest lobe and is commonly linked to traumatic brain injuries [5].

Due to the extreme rarity of traumatic cerebral myiasis, there is no established standard treatment protocol. However, in cases such as this one, the removal of the maggot infestation appears to be a suitable initial step. The administration of broad-spectrum antibiotics to address any concurrent bacterial infections and prevent secondary infections is also advisable. It is important to assess the patient's mental status to gauge the level of cognitive impairment, if present, and determine their capacity to make decisions regarding their care [7].

A relatively favorable outcome is observed despite the significant cortical exposure. This may be explained by the paradoxical protective effect of larval activity. Although the patient's brain sustained structural damage as a result of the larvae's activities, it might also act as a safeguard by eliminating dead tissues, therefore stopping a further bacterial invasion of the patient's central nervous system. This, in turn, could have created an unfavorable environment for bacterial infection, ultimately contributing to a more positive outcome even in cases where treatment was delayed. Nonetheless, the involvement of large areas of the brain could end up with fatal outcomes [5].

Although brain myiasis is uncommon, it should be taken into account as a possible diagnosis for surgical wound dehiscence, particularly in underdeveloped nations where myiasis-promoting factors are present. It is important to consider this option, particularly if there are no conventional indications of inflammation [8]. Besides, it is imperative to maintain proper hygiene in these underprivileged sections of society, and primary care physicians should be trained to handle such cases.

The paucity of data related to traumatic cerebral myiasis stresses the reporting of similar cases. The present case is the 19th documented case globally and the fourth in India of cerebral myiasis and the first reported case from India of traumatic cerebral myiasis.

## Conclusions

Traumatic cerebral myiasis is an extremely rare clinical condition in humans. The first report of this from India is presented, in which an Indian male in his 50s was brought with an open frontal wound infested with maggots of *Musca domestica nebulo*. Wound cleaning with debridement, removal of the maggot infestation, and administration of broad-spectrum antibiotics were done. This case emphasizes the importance of timely management in these cases, as delay could result in damage to vital structures in the head and neck areas. Healthcare professionals should possess the knowledge and skills to identify cases and should be prepared to commence suitable supportive treatments when needed in order to reduce morbidity.
